# Use of quercetin in animal feed: effects on the P-gp expression and pharmacokinetics of orally administrated enrofloxacin in chicken

**DOI:** 10.1038/s41598-018-22354-1

**Published:** 2018-03-13

**Authors:** Zohaib Ahmed Bhutto, Fang He, Mire Zloh, Jing Yang, Jinhu Huang, Tingting Guo, Liping Wang

**Affiliations:** 1 Joint International Research Laboratory of Animal Health and Food Safety, College of Veterinary Medicine, Nanjing Agricultural University, Nanjing, 210095 PR China; 2 School of Life and Medical Sciences, University of Hertfordshire, College Lane, Hatfield, AL10 9AB UK; 3 Medical College of Yangzhou University, Yangzhou, Jiangsu Province 22500 PR China

## Abstract

Modulation of P-glycoprotein (P-gp, encoded by Mdr1) by xenobiotics plays central role in pharmacokinetics of various drugs. Quercetin has a potential to modulate P-gp in rodents, however, its effects on P-gp modulation in chicken are still unclear. Herein, study reports role of quercetin in modulation of P-gp expression and subsequent effects on the pharmacokinetics of enrofloxacin in broilers. Results show that P-gp expression was increased in a dose-dependent manner following exposure to quercetin in Caco-2 cells and tissues of chicken. Absorption rate constant and apparent permeability coefficient of rhodamine 123 were decreased, reflecting efflux function of P-gp in chicken intestine increased by quercetin. Quercetin altered pharmacokinetic of enrofloxacin by decreasing area under curve, peak concentration, and time to reach peak concentration and by increasing clearance rate. Molecular docking shows quercetin can form favorable interactions with binding pocket of chicken xenobiotic receptor (CXR). Results provide convincing evidence that quercetin induced P-gp expression in tissues by possible interaction with CXR, and consequently reducing bioavailability of orally administered enrofloxacin through restricting its intestinal absorption and liver/kidney clearance in broilers. The results can be further extended to guide reasonable use of quercetin to avoid drug-feed interaction occurred with co-administered enrofloxacin or other similar antimicrobials.

## Introduction

Quercetin is one of the flavonoids which are natural polyphenolic compounds found in numerous components of the human daily diet, including red wine, onions, apples, tea and grapefruit juice^1^. It exerts a broad range of fascinating clinical properties, such as anti-inflammatory^2^, antineoplastic^3^, antimicrobial^4^, antiallergic^5^ and antiviral^6^ activities. The quercetin is used as a phytogenic additive in chicken feed due to a wide variety of expected beneficial effects on growth performance, oxidation stability, egg and meat quality, immune characteristics and anti-inflammation^7^. In addition to those expected beneficial effects of quercetin in chicken feed, it was also demonstrated that quercetin plays a role in modulation of the permeability-glycoprotein (P-gp) and its functional activity^8^. P-gp, encoded by multidrug resistance protein 1 (Mdr1) or ATP-binding cassette sub-family B member 1 (Abcb1) gene, is the main member of ABC transporters responsible for the efflux of numerous xenobiotics^9^ and it plays a central role in absorption, distribution, and excretion of therapeutic agents^10,11^. It was demonstrated that quercetin has a clinical role in drug-drug interaction for transportation and bioavailability of P-gp substrates in various mammalian species such as pig^11^, rat^12^, rabbit^13^, and in lamb^14^. Furthermore, it was reported that quercetin induces expression of P-gp in mice^15^, and human healthy subjects^16^, while downregulates P-gp expression in gastric cancer cells^17^. These findings suggest that P-gp expression can possibly be regulated by quercetin and consequently affect the pharmacokinetics of therapeutic agents. However, considering the reported substantial interspecies differences in P-gp expression, these findings cannot be directly extrapolated from rodents and humans to chickens. The research on modulation of P-gp expression and drug efflux by quercetin in chicken is thus required.

Enrofloxacin is a frequently used fluoroquinolone in the poultry industry in China for the medication of infections caused by bacteria, rickettsiae, and mycoplasmas^18^. It has been proved that enrofloxacin is the substrate of P-gp in chicken and pigs^19,20^. Therefore, an important line of P-gp investigations is its pharmacokinetic interactions with feed or feed additives and their possible impact on the clinical efficacy of antibiotics. Herein, we investigated the effects of quercetin on P-gp expression levels and its functional activity in *in vitro* and *in vivo* model systems as well as pharmacokinetics interaction of quercetin with enrofloxacin in chicken.

Our results indicate that quercetin could upregulate the expression and activity of the P-gp in Caco-2 cells and in different tissues of the broiler, and importantly modulate pharmacokinetics of orally administered enrofloxacin in chicken. The current results can be helpful in guiding rational use of additives and drugs in the poultry industry to avoid possible adverse effects.

## Results

### Effect of quercetin on P-gp expression in Caco-2 cells

**Figure 1:**
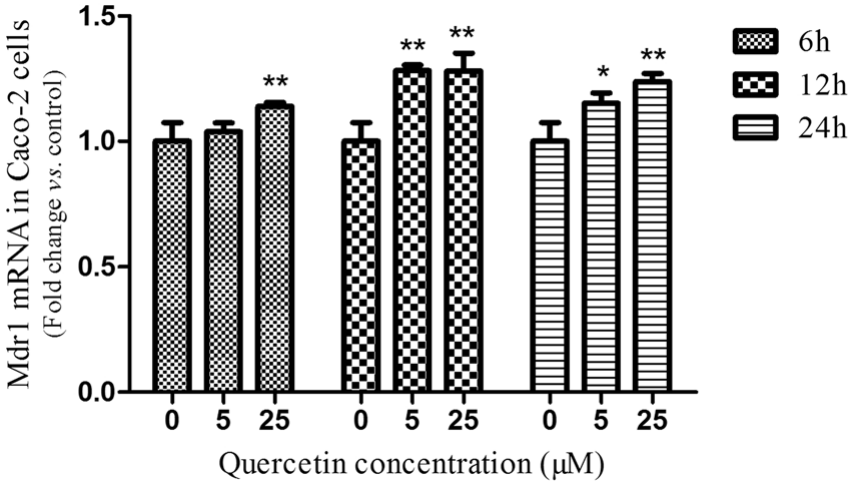
The effect of quercetin on mdr1 mRNA levels in Caco-2 cells. Each sample was run in triplicate. GADPH was used as a reference gene for normalization. Results are represented as mean ± S.E.M and were analyzed by one-tailed ANOVA using SPSS 18.0 version followed by a least significant difference (LSD) test for individual comparisons. *P < 0.05 and **P < 0.01 between quercetin treated *vs*. untreated Caco-2 cells at different time points.

Mdr1 mRNA expression levels were quantified using RT-PCR following different quercetin concentrations (5 and 25 µM) at different consecutive time points at 6 h, 12 h and 24 h (Fig. 1). When compared to control, the addition of two different concentrations of quercetin had significantly up-regulated the Mdr1 mRNA in Caco-2 cells at all time-points investigated (*P* < 0.05, *P* < 0.01), except at 6 h after the addition of 5 µM quercetin concentration.

**Figure 2:**
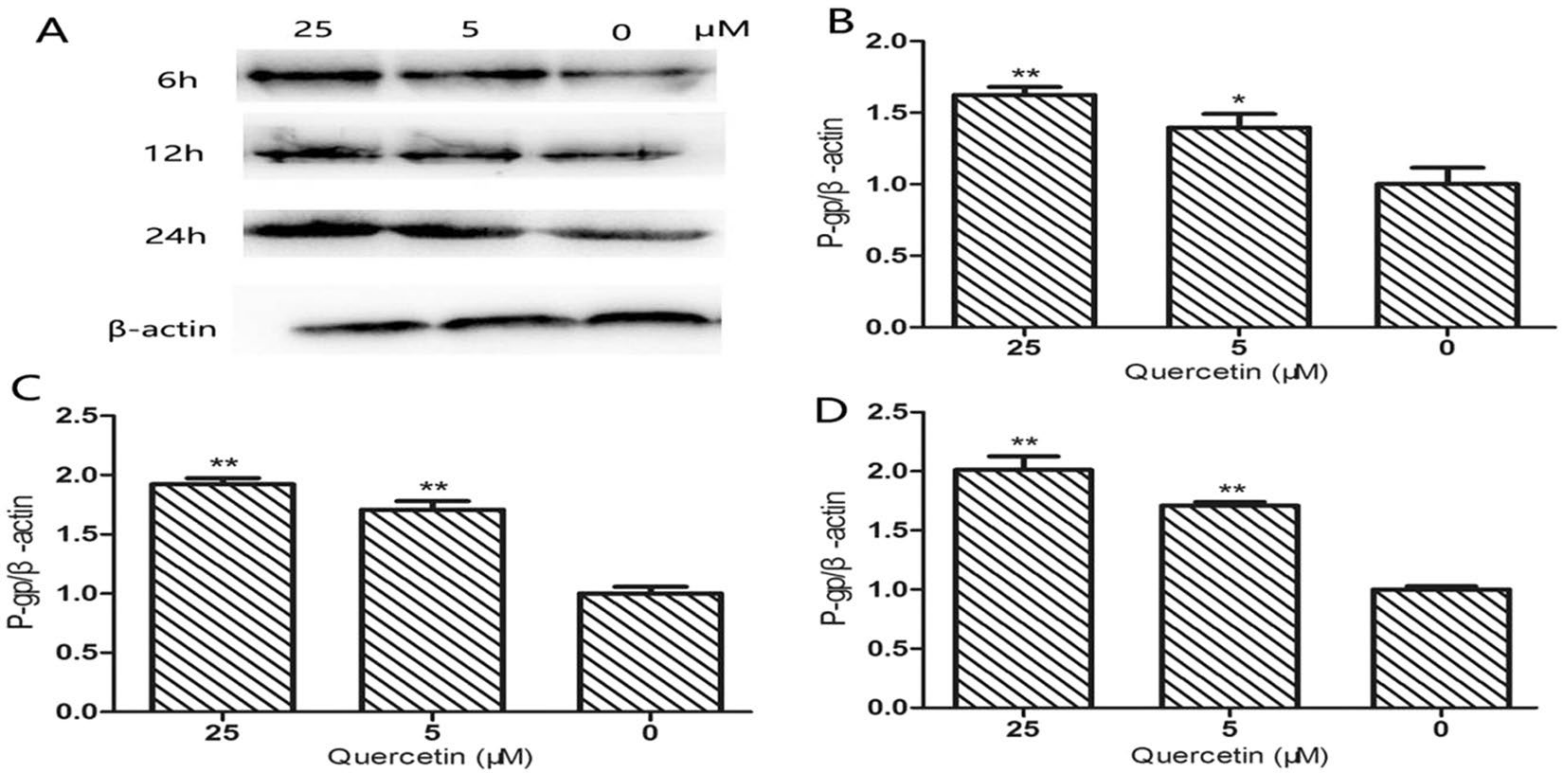
Modulation of P-gp expression in Caco-2 cells by quercetin. (**A**) Immunoblot of human P-gp protein; The relative protein expression levels different time points is shown in (**B**) 6 h, (**C**) 12 h, and (**D**) 24 h. Graphical bars show mean ± SEM (n = 3) of the P-gp/β-actin band intensities ratios. The experiments were run under similar conditions and blots were cropped from different parts of gel and original full-sized images. Samples were obtained from the same experiment and blots were processed concurrently.

The P-gp protein expression in Caco-2 cells was also examined by Western blotting at the same time points after administration of different concentrations of quercetin (Fig. 2A). The expression of the P-gp protein was significantly increased (*P* < 0.05, *P* < 0.001) at all time-points in quercetin-treated cells *vs*. untreated cells (Fig. 2B–D). The increase in P-gp protein levels was in a good correlation with the increase in mRNA levels at 6, 12 and 24 h, except for a 6 h time point after administration of 5 µM quercetin, which is not uncommon observation due to various factors that affect transcription rates and protein expression.

**Figure 3:**
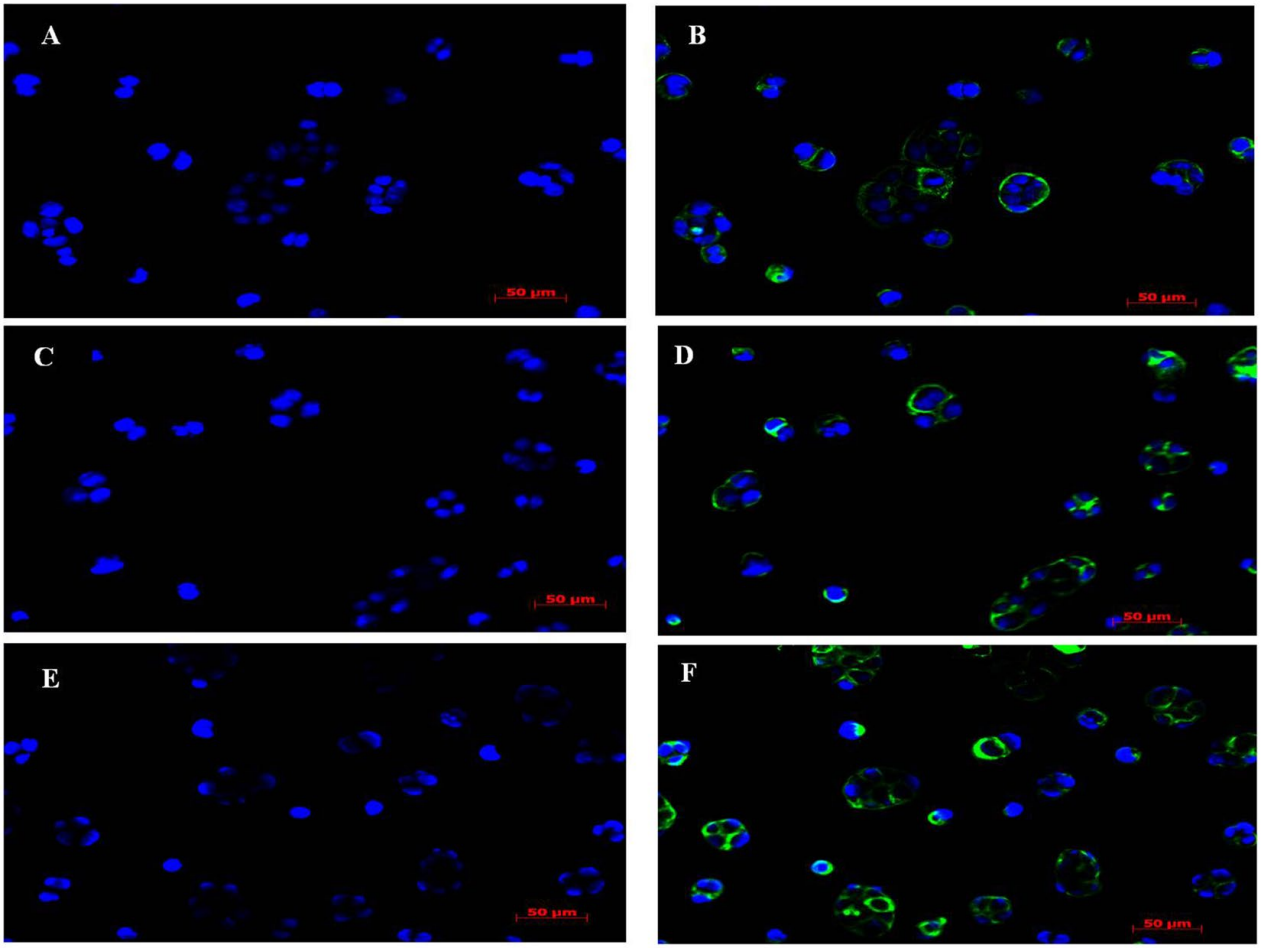
P-gp expression levels in Caco-2 cell line monitored through immunofluorescence following 24 h exposure to quercetin. The nuclei of cells counterstained by DAPI in Caco-2 cells were (**A**) not treated with quercetin (control), and treated with quercetin at (**C**) 5 µM and (**E**) 25 µM. Immunofluorescence staining shows the P-gp expressions in (**B**) cells not treated with quercetin (control) and in cells treated with quercetin at (**D**) 5 µM and (**F**) 25 µM. The immunofluorescence staining on the membrane of Caco-2 cells was much stronger in quercetin-treated cells as compared to control.

Moreover, immunofluorescence staining demonstrated an overexpressed level of P-gp protein in the cell membrane of quercetin-treated Caco-2 cells (Fig. 3D,F), and merely insignificant or negligible P-gp staining was seen in non-treated Caco-2 cells (Fig. 3B). For comparison of the immunofluorescence reactivity, the nuclei were counterstained with DAPI (Fig. 3A,C and E). The above results evidenced that P-gp protein was highly expressed in Caco-2 cells following the addition of quercetin.

### Effect of quercetin on Mdr1 mRNA expression in broilers

**Figure 4:**
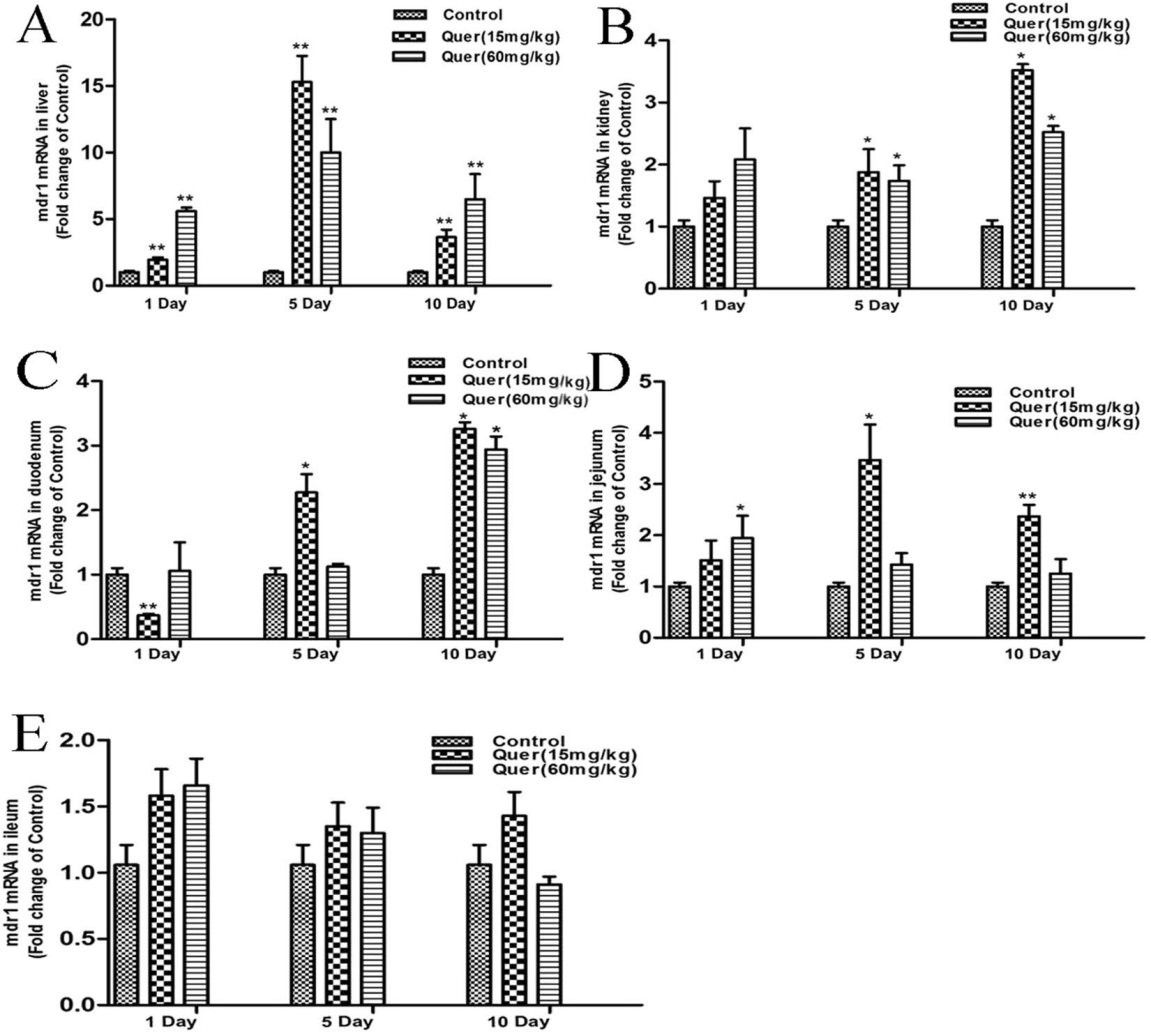
Expression levels of Mdr1 mRNA in different tissues of broilers detected by real-time RT-PCR (n = 5). Comparison of Mdr1 mRNA levels in untreated (control) and quercetin-treated adult healthy broilers on different days in (**A**) liver, (**B**) kidney, (**C**) duodenum, (**D**) jejunum and (**E**) ileum; β-actin was used as housekeeping gene for normalization. Results are represented as mean ± S.E.M and were analyzed by one-tailed ANOVA using SPSS 18.0 version followed by a least significant difference (LSD) test for individual comparisons. *P < 0.05, **P < 0.01.

The expression levels of Mdr1 mRNA were evaluated in different tissues of broilers by real-time RT-PCR after quercetin administration at different dosages (15 and 60 mg/kg) for 1, 5 and 10 days (Fig. 4). When compared to control, significant up-regulation was observed in liver Mdr1 mRNA levels in quercetin-treated groups on all consecutive days. In the kidney, Mdr1 mRNA expression significantly increased with both doses of quercetin on days 5 and 10 (*P* < 0.05). In the duodenum, Mdr1 mRNA expression levels significantly increased with 15 mg/kg concentration of quercetin for 5 and 10 days of administration, and with 60 mg/kg concentration of quercetin for 10 days of administration (*P* < 0.05). In jejunum, Mdr1 mRNA expression levels were increased with 15 mg/kg quercetin concentration on 5 and 10 days, while Mdr1 mRNA was also increased with 60 mg/kg dose of quercetin on day 1 (*P* < 0.05). In ileum, the Mdr1 expression levels increased non-significantly in all treatment groups. Amongst all tissues, the highest Mdr1 mRNA expression was seen in liver followed by kidney, duodenum, jejunum, and ileum.

### Evaluation of P-gp function by Rho 123 uptake after quercetin administration in chickens

**Figure 5:**
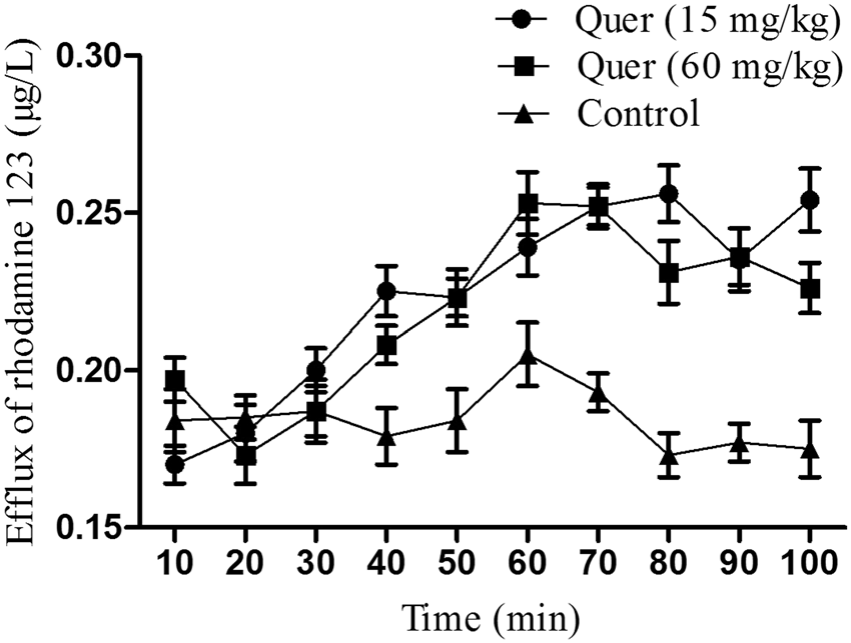
Mean Rho 123 concentrations *vs*. time curves in the jejunum of broilers. Control group I: PBS alone administered orally to adult healthy broilers (n = 6); Groups II and III: Co-administration of quercetin via the oral route (15 mg/kg, n = 6) and (60 mg/kg, n = 6), respectively. PBS and quercetin were administered for 3 days, which was followed by Rho 123 perfusion experiments for all groups. Each point represents the mean ± S.E.M of 6 broilers.

To further evaluate whether up-regulated Mdr1 mRNA expression by quercetin is accompanied by stronger efflux function in the small intestine, perfusion model in the jejunum of broilers was chosen for drug-drug interaction study (quercetin-substrate). The jejunum perfusion was evaluated by monitoring Rho 123 concentration (control and quercetin-treated samples) *vs*. time as depicted in Fig. 5. Efflux ratios in the jejunum of broilers after perfusion of Rho 123 were presented in Table 1. When compared to control, results showed an obvious increase of Rho 123 concentrations in the perfusion fluid for quercetin-treated broilers. Rho 123 absorption rate constant (Ka) was with a downward tendency but non-significantly. However, an apparent permeability coefficient (*Papp*) significantly (*P* < 0.05) decreased in quercetin-treated broilers. The concentrations of quercetin for a certain period of time induced the efflux activity of P-gp which correlates to overexpression of Mdr1 mRNA in the jejunum.

**Table 1:** Transport of Rho123 in jejunum of broilers (mean ± S.E.M, n = 6).

Parameters	Control	Quercetin (15 mg/kg)	Quercetin (60 mg/kg)
Ka (min^−1^)	0.153 ± 0.034	0.089 ± 0.008	0.084 ± 0.017
*Papp* (cm ∙ min^−1^)	0.013 ± 0.001	0.0091 ± 0.001*	0.0088 ± 0.001*

^*^P < 0.05 significant difference control *vs*. treated groups. Ka: drug absorption rate constant; *Papp*: apparent permeability coefficient.

### Pharmacokinetics assessment of enrofloxacin after quercetin administration in chicken

**Figure 6:**
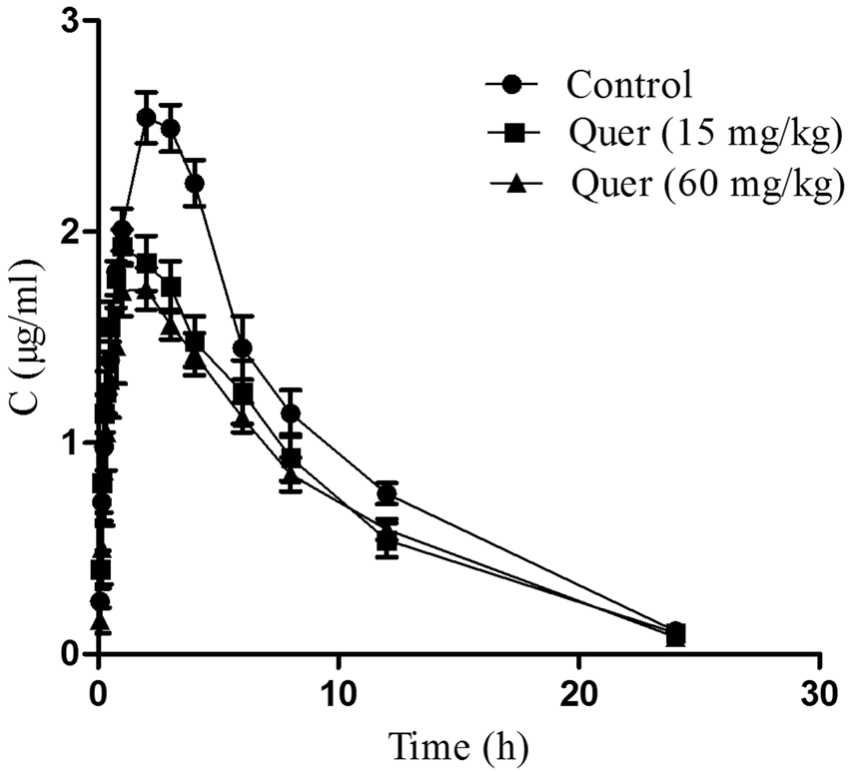
Mean plasma concentration of enrofloxacin *vs*. time curves after a single administration of the drug (10 mg/kg) without and with co-administration of quercetin at two concentrations (15 and 60 mg/kg). Each point represents the mean ± S.E.M of 5 broilers.

Following oral administration, a one-compartment model was found to provide the best fit for concentration-time results of enrofloxacin. The plasma concentrations *vs*. time curves for enrofloxacin are presented in the Fig. 6. Pharmacokinetic parameters of enrofloxacin in broilers previous to and following administration of quercetin are shown in Table 2. As compared to control group, area under curve (AUC, 17.35 ± 1.04 μg∙h/mL) and peak concentration (C_max_, 1.79 ± 0.13 μg/mL) were decreased and drug clearance rate were increased (CL, 563.45 ± 29.76 mL/h/kg) when high concentration (60 mg/kg) of quercetin was administered (*P* < 0.05). The decrease of time to reach peak concentration (T_max_, 1.40 ± 0.24 and 1.60 ± 0.24 h) was observed when low and high concentrations of quercetin were co-administered (*P* < 0.01, *P* < 0.05). By administrating both doses of quercetin, the non-significantly rising trends were seen in the apparent volume of distribution (Vd). These results suggest that by administrating the quercetin can possibly induce the higher expression level of Mdr1 mRNA in healthy broilers resulting in a reduced peak concentration of enrofloxacin in the blood. This assumption supports a correlation of observed AUC decrease and Vd increase to reduced oral absorption.

**Table 2:** Pharmacokinetic parameters of oral enrofloxacin in broilers (mean ± S.E.M, n = 5).

Parameters	Control	Quercetin (15 mg/kg)	Quercetin (60 mg/kg)
T_1/2_ (h)	4.81 ± 1.06	4.94 ± 0.72	4.76 ± 0.56
T_max_ (h)	2.75 ± 0.25	1.40 ± 0.24**	1.60 ± 0.24*
C_max_ (µg/mL)	2.67 ± 0.30	2.00 ± 0.10	1.79 ± 0.13*
AUC_0–24h_ (µg·h/mL)	23.75 ± 2.03	18.31 ± 1.51	17.35 ± 1.04*
AUC_0~∞_ (µg·h/mL)	24.69 ± 2.05	19.11 ± 1.34	17.95 ± 0.98*
Vd (mL/kg)	2.83 ± 0.60	3.91 ± 0.75	3.92 ± 0.58
CL (mL/h/kg)	413.17 ± 32.80	534.80 ± 41.21	563.45 ± 29.76*

*P < 0.05, **P < 0.01 significant difference control *vs*. treated groups. T_1/2_: half-life; T_max_: the time to reach peak concentration; C_max_: the peak concentration; AUC_0–24 h_ and AUC_0~∞_: the area under the plasma concentration-time curve from zero to 24 h and infinity, respectively; Vd: apparent volume of distribution; CL: clearance rate.

### Molecular modelling

**Figure 7:**
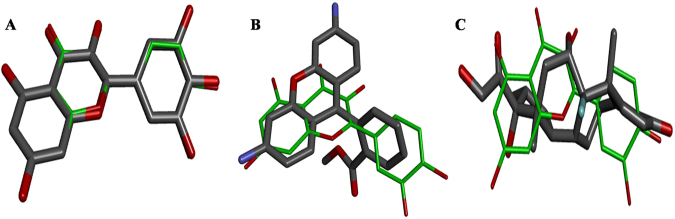
The molecular similarity of quercetin (green thin lines) to P-gp inducers (thick grey lines) (**A**) myricetin, (**B**) rhodamine 123 and (**C**) dexamethasone. All molecules are shown in stick representation colored according to CPK scheme except for quercetin carbon atoms colored in green. Hydrogen atoms are not shown for clarity.

*In silico* analyses were carried out to establish a potential mechanism of action of quercetin. The shape and features similarity calculation has shown that quercetin, as anticipated, has high similarity to a range of flavonoids that are known to induce expression of P-gp^15^ (Table 3). The hybrid score ranges from around 1.7 for similarity to chrysin and myricetin, which goes down to 1.24 for the lowest similarity to genistein. These results suggest a good overlap in the shape of similar molecules as exemplified by shape complementarily between quercetin and myricetin (Fig. 7).

**Table 3:** The molecular similarity and docking scores of quercetin obtained by calculating shape and features similarity of quercetin to known P-gp inducers and by predicting binding affinities to the chicken xenophobic receptor (CXR) and chicken vitamin D receptor (CVDR).

Name	Molecular similarity to quercetin expressed as Hybridscore	Docking scores against protein target (kcal/mol)	
		CXR	CVDR
quercetin	2.00	−8.20	−7.80
chrysin	1.70	−8.20	−8.20
myricetin	1.66	−8.00	−7.90
taxifolin	1.50	−8.10	−7.70
cyanidin	1.36	−7.90	−7.50
isoxanthohumol	1.34	−9.70	−9.00
genistein	1.24	−7.80	−7.70
rhodamine123	1.00	−9.80	−9.70
oxycodone	0.98	−8.80	−8.60
morphine	0.90	−8.10	−8.00
dexamethasone	0.86	−8.90	−8.10
amprenavir	0.73	−8.40	−9.40
nelfinavir	0.73	−9.80	−10.20
indinavir	0.71	−9.10	−8.60
saquinavir	0.68	−10.00	−9.80
retinoicacid	0.67	−8.60	−7.80
rifampin	0.65	13.10	13.90
vincristine	0.63	10.50	12.50
ritonavir	0.61	−8.80	−8.70
cyclosporinA	0.42	105.50	120.20
clotrimazole	—	−10.90	−10.00
phenothiazine	—	−7.20	−6.50

**Figure 8:**
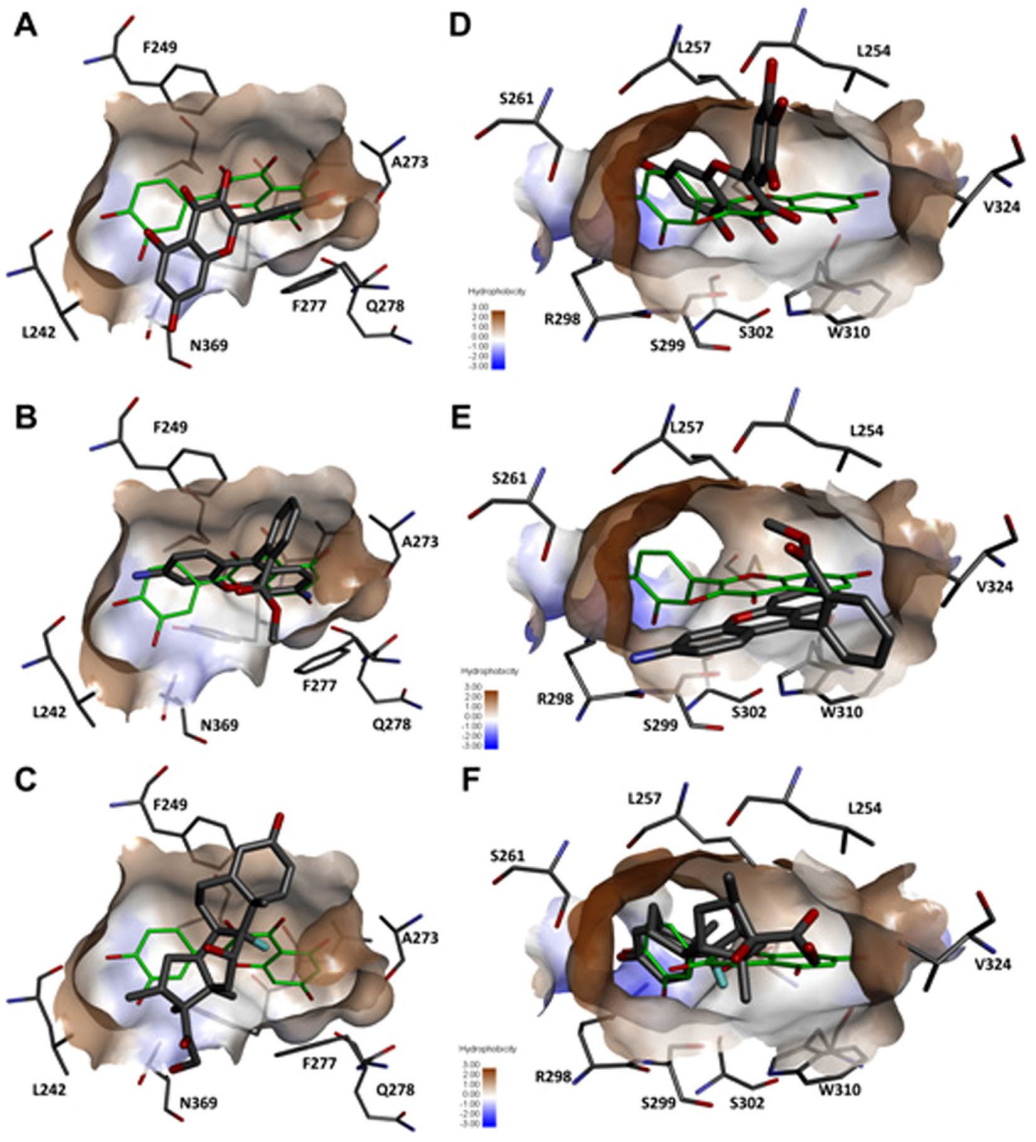
Molecular docking solutions against CXR and CVDR targets obtained for quercetin (green thin lines) and p-gp inducers (thick grey lines). Quercetin docking poses are compared to docking poses of myricetin, rhodamine 123 and dexamethasone in the active site of the CXR (**A**–**C**) and CVDR (**D**–**F**). Active sites are delineated by the hydrophobic surface and surrounding residues which are labelled and represented as thin grey lines. Hydrogen atoms are not shown for clarity.

The docking scores support our assumption that quercetin may interact with the same proteins as the other P-gp inducers, and in this case, we have focused on the chicken xenophobic receptor (CXR) and chicken vitamin D receptor (CVDR) that are known to be involved in the up-regulation of P-gp expression^15,21^. The calculated interaction energies between quercetin and homology models of these two receptors in chicken are very similar to docking scores of most P-gp inducers that can bind to both proteins (Table 3). The docking score of quercetin indicates less favorable interactions with these proteins when compared to molecules that are larger and can form more interactions with protein binding sites (e.g. isoxanthohumol, nelfinavir, saquinavir, clotrimazole). However, the quercetin can fit into a binding pocket and form favorable interactions with both proteins as both flavonoid and non-flavonoid based P-gp inducers (Fig. 8). The interaction energy between quercetin and CXR is more favorable when compared to the interaction energy between quercetin and CVDR.

## Discussion

Quercetin is found in a broad range of fruits, vegetables, and beverages and may lead to possible pharmacoepigenetic interactions as previously indicated^22^. As one of the ingredients in poultry feed that comes from plants, quercetin may affect the bioavailability or the withdrawal period of P-gp substrates in chickens, which is of high practical relevance from therapeutic efficacy and food safety point of the view. The results of our study suggest that quercetin can alter the expression levels and functional activity of P-gp, and therefore it is involved in natural product-drug interaction when simultaneously administered with P-gp substrate enrofloxacin. To the best of our knowledge, this is the first report to demonstrate that quercetin could up-regulate Mdr1 expression and its efflux function in chicken.

No suitable cell-based model systems (passage cells) or effective antibody of chicken P-gp pose one of the most difficult obstacles for studying the chicken P-gp and effects of small molecules on its function. Caco-2, a human colorectal adenocarcinoma cell line, has been accredited by the US Food and Drug Administration (FDA) for *in vitro* transport and P-gp expression studies^22,23^. In this study, Caco-2 cell line was firstly selected as an *in vitro* model for validating the effects of quercetin on P-gp expression at the transcriptional and translational level. These results established that quercetin is a potential inducer of P-gp mRNA/protein, which was consistent with some previous reports about flavonoids inducing the P-gp expression^15,24,25^. Therefore, the *in vivo* tests in chicken were undertaken. All results of this study corroborated that quercetin can upregulate the Mdr1 expression in chicken in line with Caco-2 cell and previous studies in human, mice, and pig^12,15,16^. Though the expression level of P-gp in protein level was not measured due to the lack of relevant chicken antibody, the increased efflux activity of chicken intestinal P-gp was further established by *in situ* experiment using Rho 123 as probe substrate. In this study, the expression level of P-gp mRNA was found to be highest in jejunum; therefore, *in situ* perfusion was performed in the jejunum. This was also in agreement with experiments and observations in previously published studies, where jejunum was chosen as a model for intestinal perfusion experiments^26,27^.

The data also confirmed that decrease in C_max_ and AUC_0–24 h_ of enrofloxacin in quercetin-treated broilers corresponded to Mdr1 mRNA overexpression. It is likely that the increased Mdr1 expression in small intestine, liver, and kidney of healthy broilers can reduce the absorption of orally administered enrofloxacin and it facilitated the clearance from bile and urine, thus lowering the systemic concentrations. The modulation of Mdr1 expression caused noteworthy changes in the pharmacokinetic profiles of enrofloxacin, which is coinciding with cyclosporine and talinolol pharmacokinetics profile due to quercetin modulation of P-gp in pig and rats and human, respectively^12,16^. The above observations appear to be contradictory to one of the studies in rat, where the increase of paclitaxel bioavailability was observed when the low doses of quercetin (2, 10, 20 mg/kg) were administered for three days^8^. That was in line with the general expectation that inhibition of the P-gp leads to the increased drug bioavailability. However, it was not experimentally investigated whether P-gp was overexpressed or inhibited. In our study, low and high doses of quercetin (15 and 60 mg/kg) were administered for 1, 5, and 10 days. Albeit, the high concentrations increased the expression of P-gp and reduced the bioavailability of enrofloxacin, administration of lower concentration of quercetin (15 mg/kg) for a short period (1 day) does not induce P-gp overexpression but inhibits the P-gp, thus having the effect on enrofloxacin concentration in chicken that is similar to the effect observed in rat.

The reported studies regarding pharmacodynamics and pharmacokinetics properties of fluoroquinolones suggested that critical breakpoints deciding the therapeutic efficacy of these drugs are defined by Cmax/MIC ratios ≥10 and AUC/MIC ratios ≥100^28,29^. The MIC of enrofloxacin reported being 2 µg∙mL^−1^ against field isolates of *Escherichia coli*^30^ and *Mycoplasma* spp.^31^ in chicken. By using 2 µg.mL^−1^, the Cmax/MIC ratios observed after a single orally administered dose of enrofloxacin were 1.335, 1, 0.895 for two quercetin groups (15 and 60 mg/kg), and for a control, respectively. The AUC/MIC ratios were 11.875, 9.1 and 8.675, respectively. As compared with control, the Cmax/MIC ratios decreased by 24.81% and 32.95% after treatment with quercetin (15 and 60 mg/kg), respectively, while AUC/MIC ratios of enrofloxacin decreased by 22.90% and 26.94% for these two groups. Taken together, P-gp overexpression by quercetin reduced the bioavailability of enrofloxacin and its plasma concentration. This is a reason for serious concern as lower antibiotic concentration may result in a possible loss of the enrofloxacin effectiveness against poultry pathogens. Importantly, additional research is required to evaluate the use of other antimicrobials and their interaction with quercetin mediated by P-gp efflux.

It is reported that various flavonoids including quercetin regulated the P-gp expression through pregnane X receptor (PXR) and constitutive androstane receptor (CAR)^32^ in human, and recently, it is also reported that quercetin upregulated the P-gp through vitamin D receptor (VDR) dependent pathway in Caco-2 cells^25^. It was previously demonstrated that the quercetin has potential to interact with multiple targets via judicious analysis using reverse docking methods^33^. In this study, it is postulated that quercetin can regulate the Mdr1 expression through a chicken xenobiotic receptor (CXR), as well as through chicken vitamin D receptor^34^ (CVDR). Findings from molecular docking analysis show that quercetin has similar structural features to many P-gp inducers (Fig. 7) and moreover, it can favorably interact with active sites in ligand binding domains of CXR and CVDR. The interaction energy between quercetin and CXR is more favorable when compared to the interaction energy with CVDR. These analyses strongly support our assumption that quercetin regulated the P-gp expression through CXR signaling pathways.

In conclusion, we investigated the quercetin modulated Mdr1 expression in Caco-2 cells and in chicken. This is the first report demonstrated that quercetin increased the Mdr1 expression in the tissues (liver, kidney, duodenum, and jejunum) of chickens. Consequently, this resulted in the Rho 123 uptake modulation and affected the pharmacokinetics of enrofloxacin in broilers. These presented findings have strong implication that quercetin contained in animal feed may have an effect on the bioavailability of numerous xenobiotics including antimicrobials, which are P-gp substrates, via overexpression of Mdr1 and efflux by P-gp.

## Materials and Methods

### Reagents/drugs, cells, and cell culture

Dulbecco’s Modified Eagle Medium (DMEM), fetal bovine serum (FBS) and Trypsin-EDTA obtained from Gibco Company. Quercetin was obtained from Shanghai reagent industry (China). China Institute of Veterinary Drug Control (Beijing, China) donated the enrofloxacin hydrochloride drug. Rhodamine 123 (Rho 123) and MTT were purchased from Sigma (St. Louis, MO, USA). Caco-2 cell line was bought from Cellular Resource Centre of Shanghai Institute for Biological Science, Chinese Academy of Sciences (CAS). The remaining compounds utilized were standard quality from a local vendor.

Caco-2 cell line were propagated on constant cultural conditions such as 37 °C temperature, 5% CO_2_ and 95% humidity in incubator; and high glucose DMEM added with 10% FBS, penicillin (100 U/mL) and streptomycin (100 μg/mL). 0.02% EDTA- 0.25% trypsin was used for trypsinization.

### Animals

Arbor Acres (AA) chickens were bought from a commercial hatchery (Nanjing, Jiangsu China). Total 100 one-day-old AA broilers were managed under recommended circumstances of light and temperature. Feed (no antimicrobials and anticoccidials) and water were given *ad libitum*. All birds were fed according to the breeding standards approved by National Research Council (NRC). Birds use and caring protocols were approved by the Science and Technology Agency of Jiangsu province. The approval ID is SYXK (SU) 2010–0005. The protocol of the study was conducted in accordance with guidelines of the Science and Technology Agency of Jiangsu Province and Nanjing Agricultural University. All efforts were made to minimize animal’s suffering. Prior to beginning the trial at adult age, all broilers were inspected free from colibacillosis.

### The expression level of P-gp in Caco-2 cells

When the Caco-2 cells reached maximum confluence, they were harvested and plated for drug treatments. 5 × 10^4^ cells were seeded in the 24-well plates and propagated for 24 h after that DMEM was removed when cells reached up to the confluence of 80%. 0, 5 and 25 µM concentrations of quercetin were applied at consecutive timings (6, 12 and 24 h) for quantification of mRNA level. For detection of protein expression, the same number of cells grown on 15 mm slides and same quercetin treatments were applied for 24 h. Each sample repeated triplicate and 0 served as control.

The expression level of mRNA for Mdr1 was measured by qRT-PCR. Whole RNA was isolated from Caco-2 cells by Trizol method (Takara, Tokyo, Japan) following the company’s recommendations. Samples were treated with 100 U DNase I (RNase-Free, Takara, and Tokyo, Japan) at 37 °C for 30 min to make sure that the entire RNA was free from DNA contamination. Afterward, the whole RNA counted through spectrophotometer (ND-1000, Rockland, DE, USA). Ratios of optical density values (260/280 nm) of the entire preparations were ranges from 1.8 and 2.0. The integrities of each RNA sample were verified by electrophoresis on a 1.4% agarose gel. cDNAs were made and RT-PCR was carried out. Negative controls involved the omission of RNA from the RT reactions and amplification with specific primer sets to verify without DNA contamination. Specific primers for mdr1 and GAPDH in human were designed as earlier reported^35^ and commercially manufactured for RT-PCR evaluation. Human GADPH was selected as a housekeeping gene for normalization. The PCR products were sequenced to confirm the uniqueness of the amplicons. 2^−ΔΔCt^ method^36^ was employed for data analysis obtained from RT-PCR.

P-gp relative expression was measured by western blotting with minor amendments^37^. Cell protein was separated utilizing protein extraction kit (Beyotime, Haimen, China). In brief, the same quantity of membrane proteins (20 μg per lane) was used during SDS-PAGE and after that transferring was done on PVDF membranes (BioRad, USA). The membranes were blocked with 5% skim milk powder and incubated with primary antibodies e.g: Mdr-1 (Santa Cruz, Germany, 1:200) and β-actin (TransGen, China, 1: 5,000) at 4 °C overnight. Then washing of membranes was done 3 times with PBST for 15 min. Afterward, HRP-labeled secondary antibody (goat anti-mouse IgG,1:5000, Boster, Wuhan, China) was applied for 1 h at 37 °C, and same washing steps were followed for 5 times. The ECL kit was used via visualizing the bands (Vazyme, China) and image were examined using chemiluminescence imaging system (5200-Tanon, China). The statistical analysis was done via bioinformatics software Image-Pro Plus 4.1. The software-based data (densitometric values) were employed to estimate the expression level of P-gp.

The procedure of immunofluorescence staining was executed as depicted formerly with a minor alteration^38^. The 4% paraformaldehyde was used for fixing of cells and after that PBC buffer including 3% BSA was employed for blocking up to 1 h. The primary antibody (1: 200, rabbit anti-P-gp) applied for overnight at 4 °C, and afterward, cells were again applied with secondary antibody (1:200, goat anti-rabbit IgG-FITC, Fermentas, Hanover, MD, USA) for 1 h at 37 °C. Finally, visualization was done through an immune reaction using DAB stain.

### The Mdr1 mRNA expression in Chicken

35 adult healthy birds (42-days-old) averagely weighted 2 ± 0.13 kg were selected for RT-PCR and divided into seven groups (5 birds *per* group). Group-I remained as a control without any treatment. The quercetin was administered orally to II, III, and IV groups with a dose rate of 15 mg/kg b.w. and to V, VI, and VII groups with a dose rate of 60 mg/kg b.w. for 1, 5 and 10 days, respectively.

The randomly chosen birds were sacrificed through decapitation after completion of the treatment period (1, 5, and 10 days), and the tissue samples (liver, kidney, small intestine) were taken from adult healthy birds. The entire set of samples was kept at −80 °C until RT-PCR evaluation was carried out. The expression levels of mRNA for Mdr1 in liver, kidney and small intestine of broilers were measured via using qRT-PCR. All RT-PCR experiments were carried out under conditions similar to those carried out in Caco-2 cells. Specific primers used for Mdr1 and β-actin in broilers were designed as earlier reported^39^ and commercially manufactured for RT-PCR evaluation. Chicken β-actin was selected as a housekeeping gene for normalization. The PCR products were sequenced to confirm the uniqueness of the amplicons. 2 ^−ΔΔCt^ method was used for analysis of the data obtained from RT-PCR.

### *In situ* perfusion of Rho 123 in quercetin-treated chicken and HPLC assay for Rho 123

Eighteen adult healthy broilers (42-days-old) with an average weight of 2 ± 0.13 kg were selected and separated into three groups (6 birds *per* group). PBS was given to Group-I which designated as the control. Groups II and III were continuously treated with quercetin orally for 5 days with doses of 15 and 60 mg/kg b.w., respectively. The experiment for perfusion model of jejunum was done as previously described with some modifications^40^. Birds fasted for 12 h and had a free access to water. All birds were anesthetized with 20% solution of urethane administered intravenously. Laparoscopy was performed by incision of the abdomen up to 3–4 cm and jejunum was exposed for a length of 10 cm. The exposed part was wrapped with a cotton pad soaked in normal saline and afterward with aluminum foil to avoid evaporation of fluids. The upper end of the lumen was catheterized with an inflow cannula, which was joined to perfusion system. The lower end of the jejunum was also catheterized with an outflow cannula to collect intestinal effluents serially. The lumen of jejunum was flushed with K-R buffer solution pre-warmed to 40 °C at a constant flow speed of 0.2 ml/min for 30 min until the clear solution emerged. Then Rho 123 (1.5 µg /L in K-R buffer) solution was perfused at a stable flow speed of 10 ml/min for 2 min for equilibration of the intestinal segment, and then it was again perfused after every 10 min at a constant flow speed of 0.2 ml/min. After that, blank solution (K-R buffer) was perfused at a constant flow speed of 5 ml/min for 5 min. The intestinal perfusate samples were taken every ten minutes for a period of 100 min in pre-weighed 5-ml glass vials and were stored at −20 °C in the dark. At the final stage of the trial, the length of the intestinal segment was measured as well as the radius of the loop (cm). The broilers were euthanized at the end of the experiments.

The concentrations of Rho123 in perfusion samples were determined using Agilent 1200 HPLC system. The perfusates were thawed out at room temperature and centrifuged at 12 000 g for 20 min. The supernatants were separated according to concentrations of Rho 123 standard solution used in perfusion experiments (0.15, 0.3, 0.75, 1.5, 3 and 7.5 μg/L). Five samples were prepared for each concentration. The sample (0.1 ml) was added to acetonitrile then through centrifugal force organic and water phases were separated and transferred into tubes. The organic phase was treated with nitrogen stream until dried and the residue was re-suspended with mobile phase solution. 10 µl of the mixture was injected into the HPLC system. The mobile phase was composed up of 0.1 M phosphoric acid (pH was adjusted to 3 with triethylamine)/acetonitrile (84:16, v/v). The perfusion samples contents were analysed using a Waters e2695 HPLC system (Waters, Japan) and Kromasil C18 HPLC Columns (5 μm, 25 cm × 4.6 mm). The speed of the mobile phase was adjusted up to 0.8 ml/min. Ultraviolet absorbance was recorded at 507 and 572 nm.

The apparent permeability coefficient (*Papp*) and absorption rate constant (Ka) of Rho123 were quantified using given equations: (1) *K*a = (1 − C_out_ Q_out_/C_in_ Q_in_)Q/V, (2) *P*_*app*_ = −Q_in_ (C_out_ Q_out_/C_in_ Q_in_)/2πrl, where, Q_in_/Q_out_- intestinal perfusate input and output volume (mL), C_in_ and C_out_- mass concentrations of enteric importer and exporter perfusate (μg ∙ mL^−1^), Q- perfusion rate (0.2 mL ∙ min^−1^), V- volume of bowel perfusion and 2πrl- area of the mass transfer surface (cm^2^).

### Experimental model for pharmacokinetic evaluation of enrofloxacin in broilers

Pilot pharmacokinetic studies with five chicken in a group were designed and conducted according to previous studies^19,41^. Further to promising results of the pilot study fifteen adult healthy chickens (42-days-old) averagely weighted 2 ± 0.13 kg were randomly separated into 3 groups for the full study. Group I broilers were given enrofloxacin orally with a single dose of 10 mg/kg b.w. and designated as the control. Groups II and III were treated with quercetin (15 and 60 mg/kg b.w., respectively) for 5 days, and followed by a single oral administration of enrofloxacin (10 mg/kg b.w.).

The enrofloxacin was orally administered with the help of gavage and drug concentrations in the blood were measured over 24 h period. The sampling time points were chosen based evaluate drug concentrations during drug absorption phase (2~3 sampling points), equilibrium phase (at least 3 sampling points near peak concentration), and the elimination phase (4~6 sampling points), as well as taking into account that whole sampling time should cover a period until drug concentration in blood drops to 1/10 of Cmax. For HPLC determination blood samples were taken into anticoagulated tubes (heparin) from all groups after 30 min post dosage regimen of enrofloxacin at 0.083, 0.25, 0.33, 0.5, 0.75, 1, 2, 3, 4, 6, 8, 12 and 24 h. The samples were transported to the laboratory on ice boxes and centrifuged at 1500 g for 10 min. The plasma was separated and preserved at −80 °C prior to high-performance liquid chromatography calibration.

### HPLC assay for detection of enrofloxacin in plasma of broilers

The enrofloxacin from plasma was quantified via Agilent-1200 HPLC system as previously reported with a few amendments^42^. Briefly, the blood samples were thawed at room temperature and centrifuged at 2 000 g for 5 min, the supernatant (0.5 ml) was added to acetonitrile and through centrifugal force organic and water phases were separated and transferred into tubes. The organic phase was treated with nitrogen stream until dried and the residue was re-suspended with a mobile phase solution. 20 µl of the mixture was introduced into the HPLC column. The mobile phase was composed up of 0.1 M phosphoric acid (pH was adjusted to 3 with triethylamine)/acetonitrile (84:16, v/v). The enrofloxacin concentration was measured using a Waters e2695 HPLC system (Waters, Japan) with Kromasil C18 HPLC Columns (5 μm, 25 cm × 4.6 mm). The flow speed of the mobile phase was adjusted up to 1.0 ml/min. Ultraviolet absorbance was calibrated at 278 nm.

### Pharmacokinetic analysis

The practical pharmacokinetic 3p97 software (Version97, Chinese Pharmacologic Association, Beijing, China) was used to calculate pharmacokinetic parameters for each individual set of data. The Akaike’s Information Criterion was applied which is the best fit for a one compartment model^43^. All calculations of the area under the concentration-time curve (AUC_0–24 h_) were done according to the linear trapezoidal method.

### Molecular modelling

Structures of quercetin and known P-gp inducers (myricetin, taxifolin, isoxanthohumol, genistein, chrysin, cyanidin, amprenavir, clotrimazole, dexamethasone, indinavir, morphine, nelfinavir, phenothiazine, retinoic acid, rifampin, ritonavir, saquinavir, oxycodone, cyclosporin A, vincristine and rhodamine 123) were collated as canonical SMILES string from PubChem database^44^. Initial three-dimensional models of all small molecules were generated from these SMILES strings using Avogadro molecular modelling software version 1.2.0^45^. Furthermore, molecules were protonated at pH 7.4 and the lowest energy conformations were calculated using Avogadro and MMFF94 force field. The final structures were saved in a mol2 file format.

The GenPept database was used to retrieve sequences of the chicken xenobiotic receptor (CXR) and chicken vitamin D receptors (CVDR); their accession numbers were AAG18374 and AAB62579, respectively. Their sequences in the fasta file format were submitted to using I-TASSER homology modelling server^46^ to obtain three-dimensional structures. The top solutions were used to carry out further molecular modelling to prepare protein structures for molecular docking. The top identified structural analogue for both structures was the retinoid X receptor (RXR) alpha found in the PDB entry 4NQA, complexed with a DNA and a molecule PDB id 965 bound in the ligand binding domain. To ensure that the active sites in the ligand binding domains of both CXR and CVDR are not sterically hindered as a result of homology modelling, Maestro Graphic User Interface v2016–2 was used to superimpose both homology models onto the 4NQA structure. The ligand 965 was copied into the binding sites of CXR and CVDR. The steric clashes were initially removed manually by adjusting the position of the phenylalanine ring that was protruding into binding sites to prepare the complexed structures for follow-up molecular dynamics simulation. Both structures were prepared using Protein Preparation wizard by adding all hydrogen atoms and setting the protonation states of all ionizable groups in the protein for pH 7^47^. The molecular simulations were performed with Desmond and OPLS2005 force field. All models were fully solvated using an explicit solvent (SPC water model) with the box size 10 Å larger than the size of a protein in all directions using System Builder. The systems were automatically neutralized by addition of an adequate number of relevant ions. Each system was minimized until the norm of the energy gradient was <0.1 kcal/mol. Furthermore, CXR and CVDR models, with their backbone fixed, were simulated for 1 ns at 300 K under constant pressure and temperature (NPT) conditions. Final structures of all models were used for further computational studies.

Molecular similarity calculations between quercetin and other P-gp inducers were calculated using SHAFTS software^48^ and its default settings. AutoDock Vina software^49^ implemented in VegaZZ scripting environment^50^ was used for docking of quercetin and all P-gp inducers into the active sites of both CXR and CVDR. The binding site, with the size of 18 Å x 18 Å x 18 Å, was positioned in the geometrical center of the bound 965 ligands that was removed from the structure prior to docking. Exhaustiveness was set to 20, and the top nine favorable binding modes were calculated for each molecule. All images were generated using Biovia Discovery Studio 2016.

### Data analysis

Whole results were presented as means ± S.E.M. and scrutinized through one-tailed ANOVA employing SPSS 18.0 version pursued via a least-significant difference (LSD) for individual comparisons. The mRNA levels were presented as the fold change relative to the mean levels of each group. Further data were evaluated through student *t*-test for individual samples. The significance point adjusted to *P* < 0.05.

## Electronic supplementary material

Supplementary Figure
