# Supplementary material for: Use of quercetin in animal feed: effects on the P-gp expression and pharmacokinetics of orally administrated enrofloxacin in chicken
Source: Sci Rep. 2018 Mar 13;8:4400. doi: 10.1038/s41598-018-22354-1 (PMC5849680; doi:10.1038/s41598-018-22354-1)

Use of quercetin in animal feed: effects on the P-gp expression and pharmacokinetics of orally administrated enrofloxacin in chicken

Authors and Affiliations

Zohaib Ahmed Bhutto[1], Fang He[1], Mire Zloh[2], Jing Yang[1], Jinhu Huang[1], Tingting Guo[3], Liping Wang[1] *

1. Joint International Research Laboratory of Animal Health and Food Safety，College of Veterinary Medicine, Nanjing Agricultural University, Nanjing, 210095, PR China

2. School of Life and Medical Sciences, University of Hertfordshire, College Lane, Hatfield, AL10 9AB, UK

3. Medical college of Yangzhou University, Yangzhou, Jiangsu Province, 22500, PR China

*Corresponding author: Dr. Wang Liping, College of Veterinary Medicine, Nanjing Agricultural University, Nanjing, 210095, PR China. Phone: +86-025-84395573; Fax: +86-025-84398669; Email: wlp71@163.com

Supplementary Figure 1. P-gp expression affected by quercetin in Caco-2 cells. Immunoblot of human P-gp protein and beta-actin at 6, 12, and 24 hours (right to left, control and treated groups). Full immunoblots corresponding to the cropped versions shown. The samples were derived from the same experiment and that blots were processed parallel.

6 hours

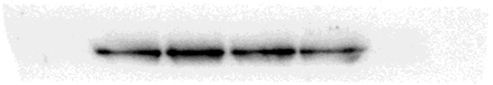

12 hours

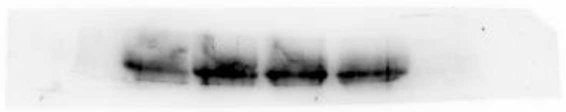

24 hours

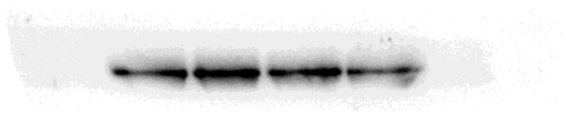

Supplement: Supplementary file 1 — Supplementary Figure [file 41598_2018_22354_MOESM1_ESM.pdf]
